# Forecasting the West Nile Virus in the United States: An Extensive Novel Data Streams–Based Time Series Analysis and Structural Equation Modeling of Related Digital Searching Behavior

**DOI:** 10.2196/publichealth.9176

**Published:** 2019-02-28

**Authors:** Abdulla Watad, Samaa Watad, Naim Mahroum, Kassem Sharif, Howard Amital, Nicola Luigi Bragazzi, Mohammad Adawi

**Affiliations:** 1 Department of Medicine B Sheba Medical Center Tel-Hashomer Ramat-Gan Israel; 2 Sackler Faculty of Medicine Tel-Aviv University Tel-Aviv Israel; 3 Zabludowicz Center for Autoimmune Diseases Sheba Medical Center Tel-Hashomer Ramat-Gan Israel; 4 NIHR Leeds Musculoskeletal Biomedical Research Unit Section of Musculoskeletal Disease, Leeds Institute of Molecular Medicine Chapel Allerton Hospital, University of Leeds Leeds United Kingdom; 5 Department of Statistics and Operations Research Tel-Aviv University Tel-Aviv Israel; 6 Department of Health Sciences Postgraduate School of Public Health University of Genoa Genoa Italy; 7 The Baruch Padeh Medical Center Zefat Israel; 8 Azrieli Faculty of Medicine Bar-Ilan University Zefat Israel; 9 Ziv Medical Center Zefat Israel

**Keywords:** forecasting model, West Nile virus, Google Trends, infodemiology, infoveillance, seasonal autoregressive integrated moving average model with explicative variable (SARIMAX)

## Abstract

**Background:**

West Nile virus is an arbovirus responsible for an infection that tends to peak during the late summer and early fall. Tools monitoring Web searches are emerging as powerful sources of data, especially concerning infectious diseases such as West Nile virus.

**Objective:**

This study aimed at exploring the potential predictive power of West Nile virus–related Web searches.

**Methods:**

Different novel data streams, including Google Trends, WikiTrends, YouTube, and Google News, were used to extract search trends. Data regarding West Nile virus cases were obtained from the Centers for Disease Control and Prevention. Data were analyzed using regression, times series analysis, structural equation modeling, and clustering analysis.

**Results:**

In the regression analysis, an association between Web searches and “real-world” epidemiological figures was found. The best seasonal autoregressive integrated moving average model with explicative variable (SARIMAX) was found to be (0,1,1)x(0,1,1)4. Using data from 2004 to 2015, we were able to predict data for 2016. From the structural equation modeling, the consumption of West Nile virus–related news fully mediated the relation between Google Trends and the consumption of YouTube videos, as well as the relation between the latter variable and the number of West Nile virus cases. Web searches fully mediated the relation between epidemiological figures and the consumption of YouTube videos, as well as the relation between epidemiological data and the number of accesses to the West Nile virus–related Wikipedia page. In the clustering analysis, the consumption of news was most similar to the Web searches pattern, which was less close to the consumption of YouTube videos and least similar to the behavior of accessing West Nile virus–related Wikipedia pages.

**Conclusions:**

Our study demonstrated an association between epidemiological data and search patterns related to the West Nile virus. Based on this correlation, further studies are needed to examine the practicality of these findings.

## Introduction

West Nile virus, first isolated in Uganda in 1937, is a widely distributed arbovirus belonging to the *Flavivirus* genus and to the *Flaviviridae* family that can cause West Nile fever. This mosquito-borne infection has a seasonal trend with peaks during summer and autumn. In 70% to 80% of West Nile fever cases, no or few symptoms are reported [1]. Symptomatic infections generally consist of self-limited influenza-like illness with high-degree fever, chills, myalgia, and arthralgia, which last for approximately 5 days. Although rare at a rate of approximately 1%, neurological diseases, such as meningitis, encephalitis, meningoencephalitis, or poliomyelitis, can occur and are of great concern because they are characterized by many sequelae, especially among elderly hospitalized patients [2].

West Nile virus was detected in North America for the first time in August 1999 during an outbreak that occurred in College Point, Queens, in New York City. A cluster of human encephalitis cases, all residing in the same 16-square-mile area, was identified by Drs Deborah Asnis (a local physician based in Queens), Marcelle Layton, and Annie Fine (of the New York City Department of Health) [2]. This cluster was preceded by anecdotal reports of dead animals and birds at the Bronx Zoo, including American crows (*Corvus brachyrhynchos*), Chilean flamingos (*Phoenicopterus chilensis*), and a snowy owl (*Bubo scandiacus*). New human cases were subsequently diagnosed in Brooklyn, the Bronx, and Manhattan. Since then, West Nile virus has spread to the contiguous states, from the Mississippi River to the Pacific Coast, causing further outbreaks such as those that occurred during the summer of 2002. Cases decreased from 2008 to 2011, but in 2012 a major outbreak took place with its epicenter in the Dallas-Fort Worth, Texas metroplex [3,4]. The Texas Public Health authority issued a public health emergency, which attracted a lot of media coverage and public opinion reaction.

The widespread resurgence of human West Nile virus disease in 2012 following several years of relatively low incidence has highlighted the continued public health hazard posed by West Nile virus, and has emphasized the need for more accurate predictive models of when and where new West Nile virus outbreaks will occur.

Web-based tools are emerging as remarkable sources of data, especially for infectious diseases, by enabling Web search monitoring in real time and potentially capturing epidemiologically relevant information [5-7]. *Infodemiology* (a portmanteau of information and epidemiology) and *infoveillance* (a portmanteau of information and surveillance) indicate the emerging “science of distribution and determinants of information in an electronic medium, specifically the internet, or in a population, with the ultimate aim to inform and improve public health and public policy” [8]. Systematically tracking and monitoring, collecting, and analyzing health-related demand data generated by novel data streams could have the potential to predict events relevant for public health purposes, such as epidemic outbreaks, as well as to investigate the effect of media coverage in terms of potential distortions, misinformation, and biases—the so-called “epidemics of fear” [9].

Little is known about West Nile virus–related digital behavior. To the best of our knowledge, only a few authors have investigated this topic. Carneiro and Mylonakis [10] reported a preliminary qualitative observation about a positive correlation between Google searches and West Nile virus epidemiological cases from 2004 to 2008. They found that the search volume exhibited a cyclical pattern, with regular peaks in August each year, reproducing the epidemiological figures. Also, Web searches related to West Nile fever symptoms (fever, headache, fatigue, rash, and eye pain) were characterized by seasonal patterns. The authors noticed increases in search volume for rash starting in May, just a month before the increases in cases. Furthermore, the top-ranked US cities in terms of West Nile virus–related search volumes were in states characterized by the highest epidemiological burden.

Bragazzi and collaborators [11] assessed the association between Web searches and cases in Italy from a quantitative standpoint from 2004 to 2015. They found a correlation of *r*=.76 (*P*<.001) and *r*=.80 (*P*<.001) between Google searches and “real-world” epidemiological cases in the same study period on a monthly basis and a yearly basis, respectively. The presence of a regular pattern in West Nile virus–related Web queries was confirmed by the partial autocorrelation function analysis and by spectral analysis. From a geospatial point of view, correlation between digital behavior and epidemiological figures yielded *r*=.54 (*P*<.05).

However, the potential predictive power of West Nile virus–related Web searches has not yet been explored. To fill this gap in knowledge, we conducted this study.

## Methods

West Nile virus–related data were retrieved, downloaded, and analyzed from several novel data streams, including Google Trends, WikiTrends, YouTube, and Google News, as well as from epidemiological repositories.

### Novel Data Streams

Google Trends (an open source tool) was mined from inception (2004) to 2015, by searching for West Nile virus in the United States and using the “search topic” option. This strategy enables one to systematically collect all the searches related to a given keyword or list of keywords (in this case, West Nile virus), including synonyms and related terms, not just the precise string of characters typed by users [12]. WikiTrends is a freely available tool that could be used to investigate information seeking behavior concerning West Nile virus. It was mined from inception (2008) to 2015. The viewing of YouTube videos was investigated from 2008 to 2015, using Google Trends and selecting “YouTube” option. Finally, Google News is an open source news aggregator that can be used to explore the media coverage of a given topic. The consumption of West Nile virus–related news was explored from 2008 to 2015 using Google Trends and selecting “Google News” option. For further details concerning novel data streams, the reader is referred to Bragazzi et al [12].

### Epidemiological Repositories

Epidemiological data related to West Nile virus cases in the United States were obtained from the Centers for Disease Control and Prevention (CDC) website and the bulletins of the *Morbidity and Mortality Weekly Report*, a publication of the CDC (data available on a trimester basis).

### Statistical Analysis

Novel data streams-generated data were retrieved and downloaded from 2004 for Google Trends and 2008 for the other open source tools to 2015. All data were analyzed on a trimester basis. To detect a potential association with “real-world” epidemiological figures, regression analyses (with time as the confounding variable) were carried out. Furthermore, novel data streams-generated data were modeled as a time series and analyzed using time series analyses. In particular, a seasonal autoregressive integrated moving average model with explicative variable (SARIMAX) was used. By visually inspecting the autocorrelogram and the partial autocorrelogram based on the autocorrelation and partial autocorrelation function, respectively, *p* (the order of the model or, in other words, the number of time lags), *d* (the degree of differencing of the model), *q* (the order of the moving average model), *P* (the order of the seasonal part of the model), *D* (the degree of differencing of the seasonal part of the model), and *Q* (the order of the moving average model for the seasonal part) coefficients and *s* (lag parameter) were determined. The explicative variable was the number of West Nile virus cases. Different models were run, and the best one was chosen based on the Akaike information criterion (AIC), corrected AIC, and Schwartz Bayesian information criterion values. The best model was used to forecast Google Trends–based relative search volumes for 2016. Furthermore, structural equation modeling and clustering analysis were used to capture the complex interplay between the different novel data streams.

Regression and clustering analyses were performed using SPSS version 24.0 (IBM Corp, Armonk, NY, USA), whereas the SARIMAX models and the structural equation modeling were carried out with XLSTAT (Addinsoft, Paris, France). A *P*<.05 was considered statistically significant.

## Results

**Figure 1:**
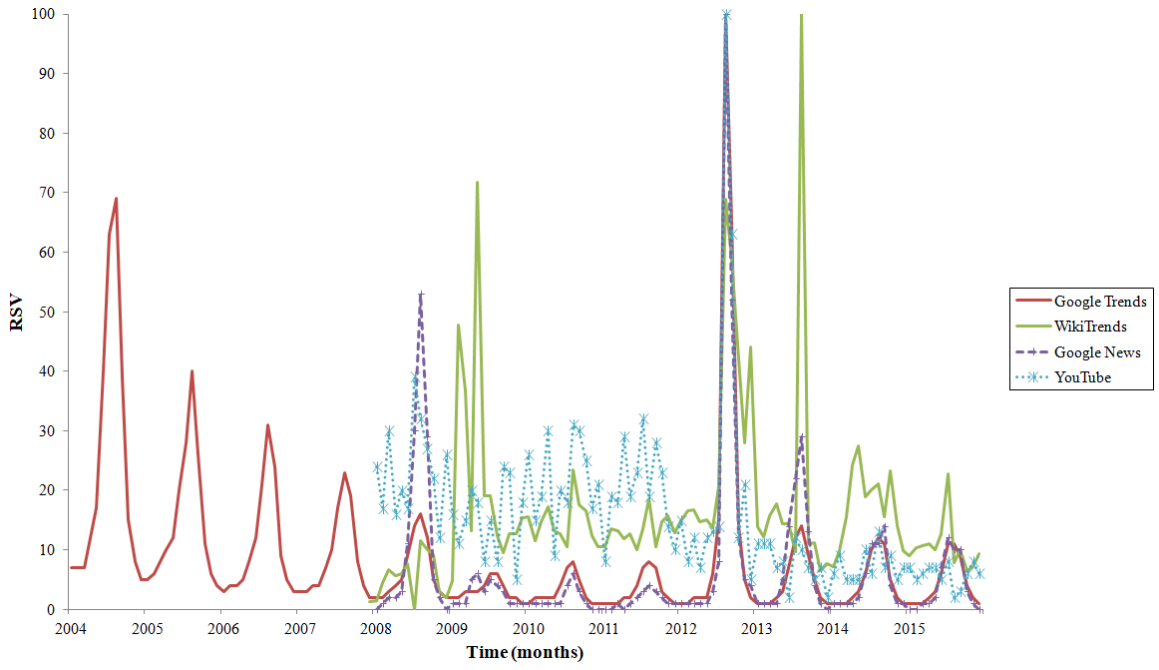
Temporal pattern of searching behavior related to the West Nile virus in the United States, as captured by four different novel data streams: Google Trends, WikiTrends, Google News, and YouTube. RSV: relative search volume (expressed as percentage).

Visual inspection of novel data streams–based data showed that each tool captured a specific digital behavior, generating specific curves which were not perfectly superimposable (Figure 1). 

**Figure 2:**
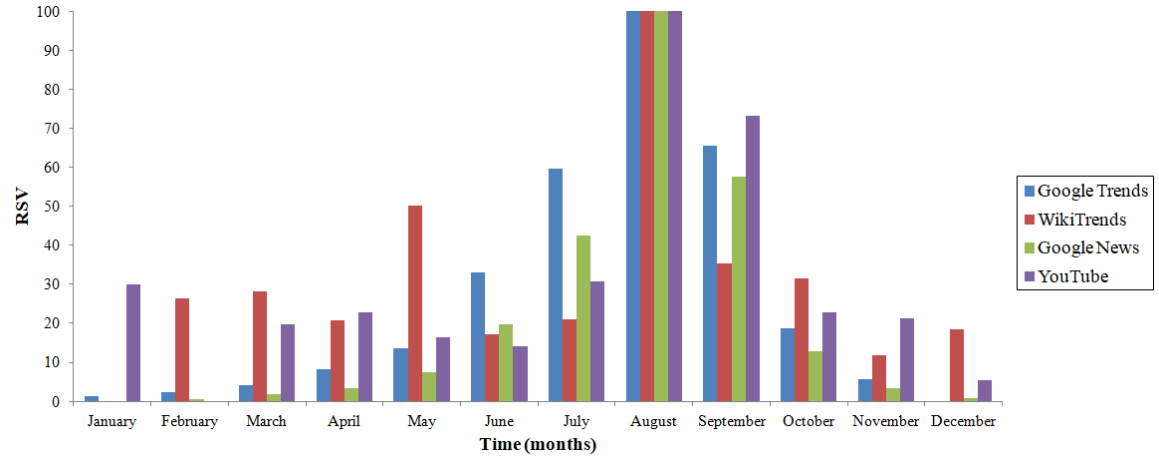
Seasonal pattern of searching behavior related to the West Nile virus in the United States, as captured by four different novel data streams: Google Trends, WikiTrends, Google News, and YouTube. RSV: relative search volume (expressed as percentage).

Concerning temporal trends, only West Nile virus–related Web searches pattern well-reproduced the epidemiological trend, with most Google queries concentrated in August. For instance, the number of accesses to the West Nile virus–related Wikipedia page (as captured by WikiTrends) and the consumption of YouTube videos exhibited high search volumes also during winter months compared to Google Trends (Figure 2).

**Figure 3:**
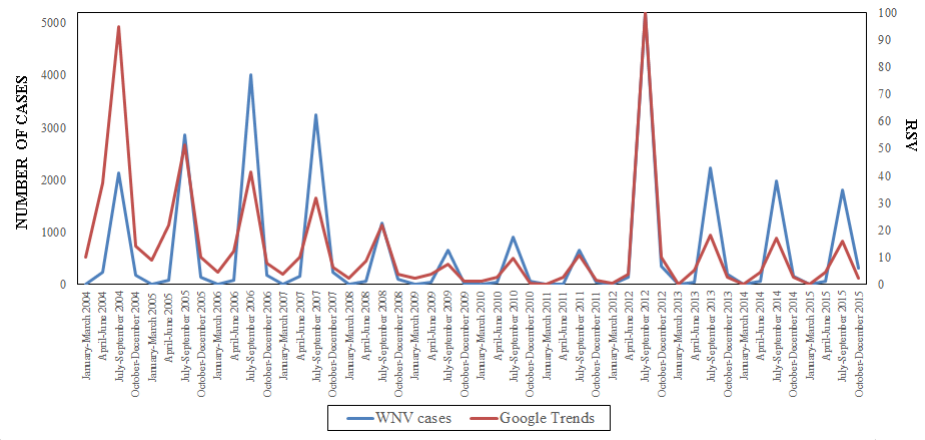
Correlation between real-world epidemiological figures of West Nile virus (WNV) cases and digital searches. RSV: relative search volume (expressed as percentage).

Regression analyses showed a significant correlation between real-world epidemiological data and novel data streams-generated figures only for Google Trends data (Table 1 and Figure 3), with the effect of year (*P*=.001) and of West Nile virus cases (*P*<.001) reaching statistical significance.

**Table 1:** Regression analyses to detect potential association between novel data streams (Google Trends, WikiTrends, Google News, and YouTube) and real-world epidemiological figures.

Source		Regression coefficient	SE	95% CI	*t*_31_	*P* value
**Google Trends**						
	Intercept	3327.876	966.213	1380.603, 5275.150	3.444	.001
	Trimester	–0.531	1.535	–3.624, 2.561	–0.346	.73
	Year	–1.653	0.481	–2.622, –0.684	–3.438	.001
	West Nile virus cases	0.014	0.001	0.011, 0.017	9.629	<.001
**WikiTrends**						
	Intercept	–7402.427	4130.337	–15863.039, 1058.184	–1.792	.08
	Trimester	1.715	4.288	–7.068, 10.498	0.400	.69
	Year	3.694	2.053	–0.512, 7.900	1.799	.08
	West Nile virus cases	–0.003	0.005	–0.012, 0.007	–0.566	.58
**Google News**						
	Intercept	8875.080	3807.169	1076.447, 16673.712	2.331	.03
	Trimester	1.478	3.952	–6.618, 9.574	0.374	.71
	Year	–4.407	1.893	–8.284, –0.530	–2.328	.03
	West Nile virus cases	–0.001	0.004	–0.010, 0.008	–0.237	.81
**YouTube**						
	Intercept	3297.355	3606.758	–4090.754, 10685.464	0.914	.37
	Trimester	3.454	3.744	–4.216, 11.124	0.923	.36
	Year	–1.622	1.793	–5.295, 2.051	–0.904	.37
	West Nile virus cases	–0.002	0.004	–0.010, 0.007	–0.453	.65

**Figure 4:**
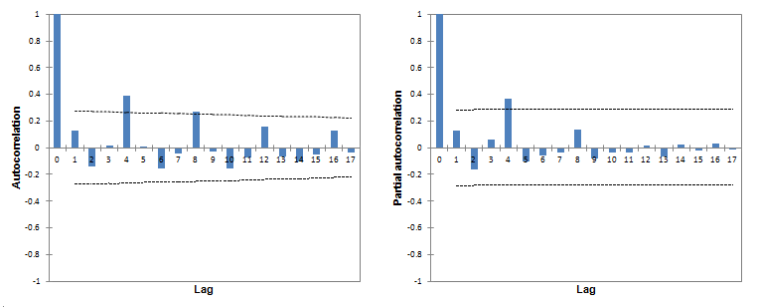
Autocorrelogram and partial autocorrelogram of West Nile virus–related search volumes generated on Google Trends.

**Figure 5:**
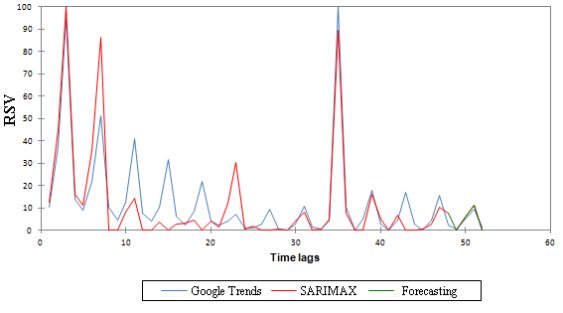
The outcome of the best seasonal autoregressive integrated moving average model with explicative variable (SARIMAX) forecasting the West Nile virus in the United States using Google Trends-generated data. RSV: relative search volume (expressed as percentage).

A Google Trends–based autocorrelogram and partial autocorrelogram are reported in Figure 4. These show statistically significant positive spikes for lags 0, 4, and 8 and lags 0 and 4, respectively. Descriptive statistics for Google Trends-generated data modeled as a time series is shown in Table 2. The best SARIMAX model was found to be (0,1,1)x(0,1,1)_4_ (Multimedia Appendix 1 and Figure 5), or a “seasonal exponential smoothing” model, being MA(1)xSMA(1). This kind of model represents a variation of the seasonal random trend, with a fine tuning obtained adding the MA(1) and the SMA(1) components. Its parameters are reported in Table 3.

**Figure 6:**
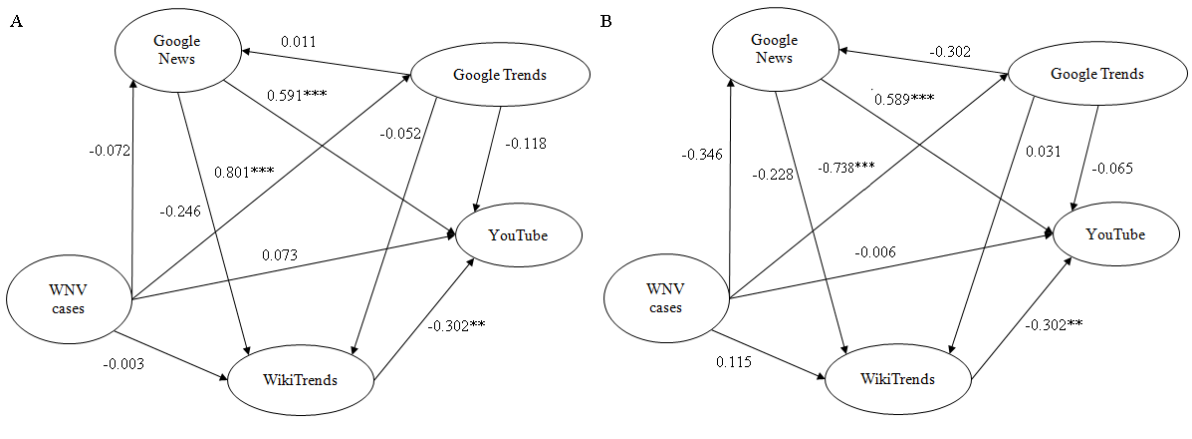
Structural equation model showing the interplay between the different novel data streams concerning West Nile virus–related searching behavior: (a) not adjusted and (b) adjusted for time as confounding variable.

Concerning structural equation modeling, the consumption of West Nile virus–related news fully mediated the relationship between Google Trends and the consumption of YouTube videos, as well as the relation between this latter variable and the number of West Nile virus cases. Web searches as captured by Google Trends fully mediated the relation between West Nile virus cases and the consumption of YouTube videos, as well as the relation between epidemiological data and the number of accesses to the West Nile virus–related Wikipedia page as captured by WikiTrends (Figure 6a). When adjusting for time as a potential confounding factor (Figure 6b), the consumption of YouTube videos mediated by the consumption of news was found to increase throughout time in a statistically significant way, although when mediated by the number of accesses to the West Nile virus–related Wikipedia page as captured by WikiTrends tended to decrease. Interestingly, the West Nile virus–related Web search behavior decreased over time (as captured by Google Trends and mediated by the number of epidemiological cases).

**Figure 7:**
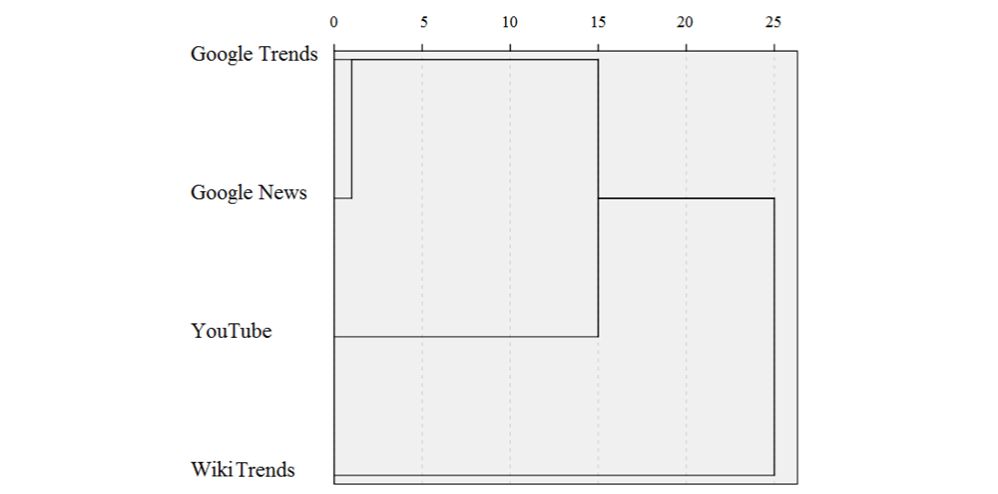
Dendrogram analysis of the four novel data streams (Google Trends, Google News, YouTube, and WikiTrends). Units are arbitrary.

Clustering analysis showed that the consumption of news was most similar to the Web searches pattern (as captured by Google Trends), which was less close to the consumption of YouTube videos and least similar to accessing the West Nile virus–related Wikipedia page as captured by WikiTrends (as can be seen by the dendrogram in Figure 7).

**Table 2:** Descriptive statistics of the Google Trends–generated data concerning Web queries related to the West Nile virus.

Lag	Autocovariance	Autocorrelation	SE	95% CI	Partial autocorrelation	SE	95% CI
0	430.22	1.00	0.00	Ref^a^	1.00	0.00	Ref
1	53.83	0.13	0.14	–0.27, 0.274	0.13	0.14	–0.28, 0.28
2	–60.95	–0.14	0.14	–0.27, 0.271	–0.16	0.14	–0.28, 0.28
3	7.85	0.02	0.14	–0.27, 0.268	0.06	0.14	–0.28, 0.28
4	166.97	0.38	0.14	–0.27, 0.265	0.37	0.14	–0.28, 0.28
5	3.73	0.01	0.13	–0.26, 0.262	–0.11	0.14	–0.28, 0.28
6	–66.73	–0.16	0.13	–0.26, 0.259	–0.06	0.14	–0.28, 0.28
7	–19.78	–0.05	0.13	–0.26, 0.256	–0.04	0.14	–0.28, 0.28
8	114.81	0.27	0.13	–0.25, 0.253	0.14	0.14	–0.28, 0.28
9	–13.47	–0.03	0.13	–0.25, 0.250	–0.08	0.14	–0.28, 0.28
10	–66.81	–0.16	0.13	–0.25, 0.247	–0.04	0.14	–0.28, 0.28
11	–30.33	–0.07	0.12	–0.24, 0.243	–0.04	0.14	–0.28, 0.28
12	67.99	0.16	0.12	–0.24, 0.240	0.01	0.14	–0.28, 0.28
13	–27.40	–0.06	0.12	–0.24, 0.237	–0.07	0.14	–0.28, 0.28
14	–45.30	–0.11	0.12	–0.23, 0.233	0.02	0.14	–0.28, 0.28
15	–22.67	–0.05	0.12	–0.23, 0.230	–0.02	0.14	–0.28, 0.28
16	56.78	0.13	0.12	–0.23, 0.226	0.03	0.14	–0.28, 0.28
17	–15.62	–0.04	0.11	–0.22, 0.223	–0.01	0.14	–0.28, 0.28

^a^Ref: reference.

**Table 3:** Parameters of the best seasonal autoregressive integrated average model with explicative variable (SARIMAX) for forecasting West Nile virus in the United States using Google Trends–generated data.

Parameter	Value	Hessian SD	95% CI	Asymptotic SD	95% CI
Constant	4.261	Ref^a^	Ref	Ref	Ref
West Nile virus cases	0.022	0.055	–0.086, 0.130	Ref	Ref
MA^b^(1)	–0.867	0.101	–1.065, –0.670	0.124	–1.110, –0.624
SMA^c^(1)	0.672	0.120	0.436, 0.907	0.150	0.379, 0.965

^a^Ref: reference.

^b^MA: nonseasonal component.

^c^SMA: seasonal component.

## Discussion

### Principal Findings

Currently, arboviruses are re-emerging infectious agents. This is not a new phenomenon—it has been happening for centuries—but today arboviral re-emergence and dispersion are more rapid and geographically extensive mainly due to globalization and to arthropod adaptation to its effects [13].

In the existing scholarly literature, different predictive models of West Nile virus have been reported. Kala and colleagues [14] described a geographically weighted regression modeling of West Nile virus risk based on environmental parameters (ie, stream density, road density, and land surface temperature) being statistically superior to classical approaches relying on ordinary least squares regression analyses in terms of predictive power. Shand and coworkers [15] improved the performance of the mosquito-based surveillance approach by incorporating rainfall, temperature, and interaction terms between precipitation and temperature as predictors. Rochlin and coauthors [16] also exploited socioeconomic factors and found a higher proportion of the population with college education, increased habitat fragmentation, and proximity to West Nile virus-positive mosquito pools correlated with higher West Nile virus human risk, whereas Trawinski and Mackay [17] relied on meteorological parameters (including average, minimum, and maximum temperature values; precipitation; relative humidity; and evapotranspiration). Day and Shaman [18] used water table depth as a measure of drought and as a proxy of arboviral transmission in two peninsular Florida regions. In another investigation, Shaman and coworkers [19] exploited hydrological variables and found that wetter spring conditions and drier summer conditions predicted increased West Nile virus human risk in Colorado’s eastern plains. Ghosh and Guha [20] performed a computational neural network analysis for capturing the nonlinear complex relationship between West Nile virus epidemiology and several different variables, including temperature, precipitation, wetlands, housing age, presence of catch basins and ditches, and mosquito abatement policies. Establishing an accurate predictive model is of crucial importance in arboviral infection control.

In our work, we exploited a variable (West Nile virus–related digital behavior), which has so far not been used in predicting West Nile virus epidemiology. We explored different novel data streams (Google Trends, WikiTrends, Google News, and YouTube) concerning seeking behavior and we were able to find a statistically significant association between epidemiological figures and digital behavior only in the case of Web searches as captured by Google Trends. Furthermore, we computed the best SARIMAX model for the period of 2004 to 2015, and we were able to forecast data related to 2016. Moreover, structural equation modeling and clustering analysis have enabled us to capture the complex interplay between the different novel data streams and the West Nile virus–related digital seeking behavior.

Even if our experience suggests the usefulness of using Google Trends for predicting West Nile virus, this should be considered as a pilot study, calling for the need for making our model more accurate and reliable, and maybe incorporating other variables (eg, environmental, socioeconomic, and ecological ones). This is of fundamental importance when designing and implementing a digital system for West Nile virus surveillance, which could complement the classical one or those actually under experimentation [21,22]. The combination of Google Trends and other predictors could reach an adequate temporal concordance with the real-world epidemiological figures and, therefore, could enable nowcasting or forecasting of new West Nile virus cases.

Our study has some limitations that should be recognized. Some of the novel data streams used provide users with relative, normalized figures, and not with raw, absolute data, thus hindering further mathematical processing and statistical analysis. Another drawback is given by the fact that Google Trends captures only a portion of the entire population, namely the percentage of people using Google as their preferred search engine (although Google is the most commonly used search engine worldwide). Furthermore, we did not perform a content analysis of the West Nile virus–related material; from the existing literature, it is known to be of rather poor quality and to exhibit some degrees of inconsistencies [23,24]. For instance, Birnbrauer and colleagues [23] explored how West Nile virus risk information was portrayed from its 1999 arrival in the United States through the year 2012, analyzing 428 articles obtained through Google News. Authors identified the following themes and topics: action, conflict, consequence, new evidence, reassurance, and uncertainty, with the action frame recurring most frequently. Moreover, West Nile virus risk was found to be improperly communicated, with statistical figures generally inaccurately reported. Dubey and coworkers [24] analyzed a total of 106 West Nile virus–related YouTube videos, 79.24% of which were found to contain useful information about the disease (60.71% related to disease prevention, and 34.52% concerning news and research updates). Videos were typically uploaded by individuals (54.6%) or news agencies (41.8%), but rarely by health care agencies (3.4%). Despite the usefulness of most West Nile virus–related videos, nonuseful videos received more views, both overall and on a daily basis. Moreover, West Nile virus–related digital behavior could have been influenced and, eventually, also distorted by extrinsic variables, such as the media coverage in terms of dissemination of imbalanced and biased information [25]. Some articles have shown that Google Trends does not always match with epidemiological data [25,26], such as in the case of Google Flu Trends [27], even though it is feasible to exploit some statistical techniques to externally revise novel data streams-generated figures, recalibrate them, and improve their accuracy and predictive power [28,29]. As such, the field of “behavioral medicine” remains largely unexplored [30], and because traditional surveillance is plagued by intrinsic limitations, enhanced methods for identification of real-time new cases and assessment of disease patterns and trends are urgently needed [31].

### Conclusions

Statistically significant temporal correlations between West Nile virus epidemiological data and Google Trends suggest the feasibility of exploiting Google Trends as an internet-based monitoring tool. This is timely and of crucial importance given the recent re-emergence of arboviral infections. Workers in the field of public health and health authorities should be aware of the public interest and reaction to West Nile virus outbreaks in terms of Web searches. They could exploit the new information and communication technologies both for performing real-time monitoring of new population-based epidemic events and for carrying out a content analysis of the available online material, promptly replying to public concerns and correcting prejudices and inaccurate and misleading reports by disseminating high-quality information. However, based on the previously mentioned limitations of this paper, further studies are warranted to make our model more useful and practical.

## Appendix

Multimedia Appendix 1 Different tested seasonal autoregressive integrated average (SARIMAX) models for forecasting the West Nile virus in the United States using Google Trends–generated data.

## Abbreviations

AIC: Akaike information criterion
SARIMAX: seasonal autoregressive integrated moving average model with explicative variable

## References

[1] Saxena V, Bolling BG, Wang T (2017). West Nile virus. Clin Lab Med.

[2] Kleinschmidt-DeMasters BK, Beckham JD (2015). West Nile virus encephalitis 16 years later. Brain Pathol.

[3] Calisher CH (2000). West Nile virus in the New World: appearance, persistence, and adaptation to a new econiche-an opportunity taken. Viral Immunol.

[4] Williamson PC, Custer B, Biggerstaff BJ, Lanciotti RS, Sayers MH, Eason SJ, Dixon MR, Winkelman V, Lanteri MC, Petersen LR, Busch MP (2017). Incidence of West Nile virus infection in the Dallas-Fort Worth metropolitan area during the 2012 epidemic. Epidemiol Infect.

[5] Pelat C, Turbelin C, Bar-Hen A, Flahault A, Valleron AJ (2009). More diseases tracked by using Google Trends. Emerg Infect Dis.

[6] Alicino C, Bragazzi NL, Faccio V, Amicizia D, Panatto D, Gasparini R, Icardi G, Orsi A (2015). Assessing Ebola-related web search behaviour: insights and implications from an analytical study of Google Trends-based query volumes. Infect Dis Poverty.

[7] Bragazzi NL, Barberis I, Rosselli R, Gianfredi V, Nucci D, Moretti M, Salvatori T, Martucci G, Martini M (2017). How often people google for vaccination: qualitative and quantitative insights from a systematic search of the web-based activities using Google Trends. Hum Vaccin Immunother.

[8] Eysenbach G (2009). Infodemiology and infoveillance: framework for an emerging set of public health informatics methods to analyze search, communication and publication behavior on the Internet. J Med Internet Res.

[9] Eysenbach G (2002). Infodemiology: the epidemiology of (mis)information. Am J Med.

[10] Carneiro HA, Mylonakis E (2009). Google trends: a web-based tool for real-time surveillance of disease outbreaks. Clin Infect Dis.

[11] Bragazzi NL, Bacigaluppi S, Robba C, Siri A, Canepa G, Brigo F (2016). Infodemiological data of West-Nile virus disease in Italy in the study period 2004-2015. Data Brief.

[12] Bragazzi NL, Dini G, Toletone A, Brigo F, Durando P (2016). Leveraging big data for exploring occupational diseases-related interest at the level of scientific community, media coverage and novel data streams: the example of silicosis as a pilot study. PLoS One.

[13] Gould E, Pettersson J, Higgs S, Charrel R, de Lamballerie X (2017). Emerging arboviruses: why today?. One Health.

[14] Kala AK, Tiwari C, Mikler AR, Atkinson SF (2017). A comparison of least squares regression and geographically weighted regression modeling of West Nile virus risk based on environmental parameters. PeerJ.

[15] Shand L, Brown WM, Chaves LF, Goldberg TL, Hamer GL, Haramis L, Kitron U, Walker ED, Ruiz MO (2016). Predicting West Nile virus infection risk from the synergistic effects of rainfall and temperature. J Med Entomol.

[16] Rochlin I, Turbow D, Gomez F, Ninivaggi DV, Campbell SR (2011). Predictive mapping of human risk for West Nile virus (WNV) based on environmental and socioeconomic factors. PLoS One.

[17] Trawinski PR, Mackay DS (2008). Meteorologically conditioned time-series predictions of West Nile virus vector mosquitoes. Vector Borne Zoonotic Dis.

[18] Day JF, Shaman J (2008). Using hydrologic conditions to forecast the risk of focal and epidemic arboviral transmission in peninsular Florida. J Med Entomol.

[19] Shaman J, Day JF, Komar N (2010). Hydrologic conditions describe West Nile virus risk in Colorado. Int J Environ Res Public Health.

[20] Ghosh D, Guha R (2011). Using a neural network for mining interpretable relationships of West Nile risk factors. Soc Sci Med.

[21] Konrad SK, Zou L, Miller SN (2012). A geographical information system-based web model of arbovirus transmission risk in the continental United States of America. Geospat Health.

[22] Carney R, Ahearn SC, McConchie A, Glasner C, Jean C, Barker C, Park B, Padgett K, Parker E, Aquino E, Kramer V (2011). Early warning system for West Nile virus risk areas, California, USA. Emerg Infect Dis.

[23] Birnbrauer K, Frohlich DO, Treise D (2017). Inconsistencies in reporting risk information: a pilot analysis of online news coverage of West Nile Virus. Glob Health Promot.

[24] Dubey D, Amritphale A, Sawhney A, Dubey D, Srivastav N (2014). Analysis of YouTube as a source of information for West Nile Virus infection. Clin Med Res.

[25] Bragazzi NL, Alicino C, Trucchi C, Paganino C, Barberis I, Martini M, Sticchi L, Trinka E, Brigo F, Ansaldi F, Icardi G, Orsi A (2017). Global reaction to the recent outbreaks of Zika virus: insights from a Big Data analysis. PLoS One.

[26] Adawi M, Bragazzi NL, Watad A, Sharif K, Amital H, Mahroum N (2017). Discrepancies between classic and digital epidemiology in searching for the Mayaro virus: preliminary qualitative and quantitative analysis of Google Trends. JMIR Public Health Surveill.

[27] Lazer D, Kennedy R, King G, Vespignani A (2014). Big data. The parable of Google Flu: traps in big data analysis. Science.

[28] Olson DR, Konty KJ, Paladini M, Viboud C, Simonsen L (2013). Reassessing Google Flu Trends data for detection of seasonal and pandemic influenza: a comparative epidemiological study at three geographic scales. PLoS Comput Biol.

[29] Santillana M, Zhang DW, Althouse BM, Ayers JW (2014). What can digital disease detection learn from (an external revision to) Google Flu Trends?. Am J Prev Med.

[30] Ayers JW, Althouse BM, Dredze M (2014). Could behavioral medicine lead the web data revolution?. JAMA.

[31] Nuti SV, Wayda B, Ranasinghe I, Wang S, Dreyer RP, Chen SI, Murugiah K (2014). The use of google trends in health care research: a systematic review. PLoS One.

