# Supplementary material for: Forecasting the West Nile Virus in the United States: An Extensive Novel Data Streams–Based Time Series Analysis and Structural Equation Modeling of Related Digital Searching Behavior
Source: JMIR Public Health Surveill. 2019 Feb 28;5(1):e9176. doi: 10.2196/publichealth.9176 (PMC6416538; doi:10.2196/publichealth.9176)
Supplement: Multimedia Appendix 1 [file publichealth_v5i1e9176_app1.pdf]

Different tested seasonal autoregressive integrated average (SARIMAX) models for forecasting the West Nile virus in the United States using Google Trends-generated data.[a]

Model	Fitting parameter										
	SSE	MSE	RMEQ	Var RB	ΔMAPE	MAPE	-2Log(llh)	FPE	AIC	AICC	SBC
(0.0.0)(0.0.0)^4	6266.13	130.54	11.43	131.20	149.60	71.87	370.22	136.10	376.22	376.77	381.84
(0.0.0)(0.1.1)^4	4584.07	104.18	10.21	148.00	388.52	143.82	331.94	104.18	337.94	338.54	343.29
(0.0.0)(1.1.0)^4	5306.45	120.60	10.98	148.62	303.22	67.02	337.00	144.72	343.00	343.60	348.36
(0.0.0)(1.1.1)^4	4396.16	99.91	10.00	147.97	474.39	81.16	330.60	119.90	338.60	339.63	345.74
(0.0.1)(0.0.0)^4	6452.93	134.44	11.59	135.39	150.47	82.76	371.59	134.44	377.59	378.13	383.20
(0.0.1)(0.0.1)^4	6452.93	134.44	11.59	135.39	150.47	82.76	371.59	134.44	377.59	378.13	383.20
(0.0.1)(0.1.0)^4	5973.29	135.76	11.65	143.42	376.51	51.74	340.99	135.76	346.99	347.59	352.34
(0.0.1)(0.1.1)^4	4403.87	100.09	10.00	141.87	504.12	144.83	330.23	100.09	340.23	341.81	349.15
(0.0.1)(1.1.0)^4	5144.74	116.93	10.81	142.35	463.78	64.17	335.56	140.31	343.56	344.58	350.69
(0.0.1)(1.1.1)^4	4237.39	96.30	9.81	141.73	592.31	83.22	329.01	115.57	341.01	343.28	351.72
(0.1.0)(0.1.0)^4	5867.95	136.46	11.68	407.91	204.48	129.99	334.54	136.46	340.54	341.15	345.82
(0.1.0)(0.1.1)^4	7258.75	168.81	12.99	237.27	827.28	187.32	345.26	168.81	351.26	351.87	356.54
(0.1.0)(1.1.0)^4	8652.92	201.23	14.19	237.35	472.82	147.55	350.98	242.51	356.98	357.59	362.26
(0.1.0)(1.1.1)^4	7036.32	163.64	12.79	236.78	924.13	199.61	344.35	197.20	352.35	353.40	359.39
(0.1.1)(0.0.0)^4	6063.49	129.01	11.36	129.11	115.19	59.98	362.52	129.01	368.52	369.07	368.52
(0.1.1)(0.1.0)^4	5867.95	136.46	11.68	407.91	204.48	129.99	334.54	136.46	340.54	341.15	345.82
(0.1.1)(0.1.1)^4	4464.88	103.85	10.19	146.20	711.40	78.65	324.82	103.83	334.82	336.44	343.626
(0.1.1)(1.1.1)^4	5626.70	130.85	11.44	145.94	401.72	92.97	333.95	157.70	345.95	348.28	356.52
(1.0.0)(0.0.0)^4	6266.13	130.54	11.43	131.20	149.60	71.87	370.22	136.10	376.22	376.77	381.84
(1.0.0)(0.1.0)^4	5940.00	135.00	11.62	142.77	760.94	47.66	340.75	141.28	346.75	347.35	352.10
(1.0.0)(0.1.1)^4	4396.24	99.91	10.00	141.39	580.82	78.40	330.13	104.56	338.13	339.15	345.27
(1.0.0)(1.1.0)^4	5132.95	116.66	10.80	141.84	622.99	62.18	335.44	146.57	345.44	347.02	354.36
(1.0.0)(1.1.1)^4	4233.36	96.21	9.81	141.24	660.33	82.57	328.94	120.88	340.94	343.21	351.65
(1.0.1)(0.0.0)^4	6054.64	126.14	11.23	126.22	170.90	63.33	368.81	131.51	376.81	377.74	384.30
(1.0.1)(0.1.0)^4	5827.14	132.44	11.51	141.89	869.64	51.01	340.00	138.59	348.00	349.03	355.14
(1.0.1)(0.1.1)^4	4396.87	99.93	10.00	141.06	592.49	77.60	330.125	104.58	342.13	344.40	352.83
(1.0.1)(1.1.0)^4	5109.61	116.13	10.78	141.39	816.97	59.91	335.29	145.90	347.29	349.57	358.00
(1.0.1)(1.1.1)^4	4518.17	102.69	10.13	140.89	839.92	132.28	331.48	129.02	347.48	351.60	361.76
(1.1.0)(0.0.0)^4	7656.74	162.91	12.76	162.95	286.51	90.47	372.93	169.99	378.93	379.49	384.48
(1.1.0)(0.1.0)^4	8002.45	186.10	13.64	197.48	284.99	119.73	346.94	194.97	352.94	353. 56	358.22
(1.1.0)(0.1.1)^4	6105.12	141.98	11.92	196.97	1016.17	167.31	337.69	148.74	345.69	346.74	352.74
(1.1.0)(1.0.0)^4	7656.74	162.91	12.76	162.95	286.51	90.47	372.93	169.99	378.93	379.49	384.48
(1.1.0)(1.1.0)^4	7129.97	165.81	12.877	197.20	1466.47	157.38	342.90	209.45	352.90	354.52	361.71
(1.1.0)(1.1.1)^4	5921.23	137.70	11.73	196.56	1121.79	177.82	336.84	173.94	348.84	351.17	359.41
(1.1.1)(0.0.0)^4	6058.94	128.91	11.35	129.06	112.72	59.83	362.48	134.52	370.48	371.44	377.88
(1.1.1)(0.1.0)^4	5857.19	136.21	11.67	394.41	231.21	124.49	334.49	142.70	342.49	343.54	349.54
(1.1.1)(0.1.1)^4	4401.96	102.37	10.12	287.28	816.16	114.86	324.44	107.25	336.44	338.78	347.01
(1.1.1)(1.1.0)^4	23591.61	548.64	23.42	340.89	$2.32 \cdot 10^{16}$	264.85	395.04	693.02	407.04	409.37	417.60
(1.1.1)(1.1.1)^4	4227.23	98.31	9.92	144.21	969.20	91.56	323.32	124.18	339.32	343.56	353.41
(2.0.0)(0.0.0)^4	6233.30	129.86	11.40	130.20	144.64	68.35	369.98	141.15	377.98	378.91	385.47
(2.0.0)(0.1.0)^4	5930.58	134.79	11.61	142.59	1285.02	46.38	340.68	147.62	348.68	349.71	355.82
(2.0.0)(0.1.1)^4	4396.69	99.92	10.00	141.25	590.64	77.78	330.13	109.44	340.13	341.70	349.05
(2.0.0)(0.1.2)^4	3960.77	90.02	9.49	134.87	802.42	90.76	326.97	98.59	338.97	341.24	349.68
(2.0.0)(1.1.0)^4	5131.03	116.61	10.80	141.69	696.71	60.52	335.42	153.44	347.42	349.69	358.13
(2.0.0)(1.1.1)^4	4233.16	96.21	9.81	141.10	659.43	82.63	328.94	126.59	342.94	346.05	355.43
(2.0.0)(1.1.2)^4	4293.13	97.57	9.88	131.85	536.33	76.57	329.41	128.38	345.41	349.52	359.68
(2.0.0)(2.1.0)^4	3553.87	80.77	8.99	136.51	673.77	85.14	323.16	128.28	337.16	340.28	349.65
(2.0.0)(2.1.2)^4	3097.71	70.40	8.39	120.73	626.72	171.39	320.77	111.82	338.77	344.07	354.83
(2.0.1)(0.0.0)^4	6049.19	126.02	11.23	126.15	158.65	61.74	368.74	136.98	378.74	380.17	388.10
(2.0.1)(0.1.0)^4	5823.82	132.36	11.50	141.50	851.32	49.56	339.97	144.97	349.97	351.55	358.89
(2.0.1)(0.1.1)^4	12496.60	284.01	16.85	140.76	362.82	221.56	377.33	311.06	391.33	394.44	403.82
(2.0.1)(0.1.2)^4	3979.01	90.43	9.51	123.90	710.11	132.22	328.33	99.04	344.33	348.44	358.60
(2.0.1)(1.1.0)^4	5100.95	115.93	10.77	141.04	737.04	59.23	335.21	152.54	349.21	352.32	361.70
(2.0.1)(1.1.1)^4	11961.09	271.84	16.49	140.56	375.46	235.43	376.74	357.69	394.74	400.04	410.80
(2.0.1)(2.1.0)^4	13174.19	299.41	17.30	135.46	351.54	206.23	378.45	475.54	394.45	398.56	408.72
(2.0.2)(0.0.0)^4	5454.01	113.63	10.66	125.64	9144.53	139.76	368.74	123.51	380.74	382.79	391.96
(2.0.2)(0.1.0)^4	15038.27	341.78	18.49	130.27	358.39	190.81	383.68	374.33	395.68	397.95	406.38
(2.0.2)(0.1.1)^4	11333.99	257.59	16.05	129.43	393.81	178.69	375.80	282.12	391.80	395.92	406.08
(2.0.2)(0.1.2)^4	10162.13	230.96	15.20	233.13	367.90	173.30	375.16	252.95	393.16	398.45	409.22

(2.0.2)(1.1.0)^{4}	5712.31	129.83	11.39	129.83	736.05	52.86	340.76	170.82	356.76	360.88	371.04
(2.0.2)(1.1.1)^{4}	4020.16	91.37	9.56	129.16	588.29	78.06	327.64	120.22	347.64	354.31	365.48
(2.0.2)(2.1.0)^{4}	13017.06	295.84	17.20	122.48	326.24	188.17	378.03	469.87	396.03	401.33	412.09
(2.1.0)(0.0.0)^{4}	6030.30	128.30	11.33	130.60	169.81	88.85	362.38	139.71	370.38	371.33	377.78
(2.1.0)(0.1.0)^{4}	6563.43	152.64	12.35	164.64	573.86	100.00	338.96	167.53	346.96	348.01	354.00
(2.1.0)(0.1.1)^{4}	5319.15	123.70	11.12	163.86	1731.52	132.44	331.76	135.77	341.76	343.38	350.56
(2.1.0)(0.1.2)^{4}	4593.35	106.82	10.34	119.21	1058.62	113.80	332.06	117.24	344.06	346.40	354.63
(2.1.0)(1.1.0)^{4}	6194.60	144.06	12.00	164.37	1574.04	123.33	336.74	190.78	348.74	351.07	359.30
(2.1.0)(1.1.1)^{4}	12321.08	286.54	16.93	286.54	$9.33\cdot10^{16}$	200.24	375.29	379.47	389.29	392.49	401.62
(2.1.0)(1.1.2)^{4}	5387.80	125.30	11.19	120.17	1146.56	134.22	341.00	165.93	357.00	361.24	371.09
(2.1.0)(2.1.0)^{4}	4289.72	99.76	9.99	152.54	1380.72	136.43	324.90	160.22	338.90	342.10	351.23
(2.1.0)(2.1.1)^{4}	4446.53	103.41	10.17	103.41	1855.43	137.76	334.14	166.08	350.14	354.38	364.23
(2.1.1)(0.0.0)^{4}	5708.62	121.46	11.02	122.21	171.31	76.26	359.84	132.26	369.84	371.30	379.09
(2.1.1)(0.1.0)^{4}	5657.82	131.58	11.47	144.85	248.57	89.69	332.95	144.41	342.95	344.57	351.76
(2.1.1)(0.1.1)^{4}	4396.94	102.25	10.11	144.46	798.55	86.42	324.24	112.23	338.24	341.44	350.57
(2.1.1)(0.1.2)^{4}	10980.06	255.35	15.98	120.69	$7.46\cdot10^{16}$	211.24	371.17	280.26	387.17	391.41	401.26
(2.1.1)(1.0.0)^{4}	5708.62	121.46	11.02	122.21	171.31	76.26	359.84	132.26	369.84	371.30	379.09
(2.1.1)(1.1.0)^{4}	5133.04	119.37	10.93	144.66	928.86	77.20	329.43	158.09	343.43	346.63	355.76
(2.1.1)(1.1.1)^{4}	10992.92	255.65	15.99	125.51	$7.39\cdot10^{16}$	210.41	371.16	338.56	389.16	394.62	405.01
(2.1.1)(2.1.0)^{4}	3583.60	83.34	9.13	138.48	1129.63	109.93	318.23	133.85	334.23	338.47	348.32
(2.1.2)(0.0.0)^{4}	5506.99	117.17	10.82	117.23	213.34	79.71	358.47	127.59	370.47	372.57	381.57
(2.1.2)(0.1.0)^{4}	5362.59	124.71	11.17	132.86	452.83	80.45	332.37	136.88	344.37	346.70	354.94
(2.1.2)(0.1.1)^{4}	4735.93	110.14	10.49	131.93	619.81	73.40	327.23	120.88	343.23	347.47	357.32
(2.1.2)(0.1.2)^{4}	10889.45	253.24	15.91	119.81	$9.61\cdot10^{16}$	216.08	370.43	277.95	388.43	393.88	404.28
(2.1.2)(1.1.0)^{4}	5684.74	132.20	11.50	132.20	580.33	74.40	334.98	175.08	350.98	355.21	365.07
(2.1.2)(1.1.1)^{4}	5161.60	120.04	10.96	131.57	585.54	92.47	332.85	158.97	352.85	359.73	370.47
(2.1.2)(2.1.0)^{4}	3270.54	76.06	8.72	124.40	1278.96	108.35	316.32	122.16	334.32	339.78	350.17
(2.1.2)(2.1.2)^{4}	9226.52	214.57	14.65	87.10	$7.65\cdot10^{16}$	194.35	368.38	344.61	392.38	402.78	413.52
(3.0.0)(0.1.1)^{4}	4396.16	99.91	10.00	140.28	553.58	77.57	330.11	114.53	342.11	344.38	352.82

[a] AIC: Akaike information criterion; AICc: corrected AIC; FPE: final prediction error; MAPE: mean absolute percentage error; MSE: mean square error; RMSE: root MSE; SBC: Schwartz Bayesian criterion/Bayesian information criterion; SSE: sum of square errors.
